# Effects of enhanced productivity of resources shared by predators in a food‐web module: Comparing results of a field experiment to predictions of mathematical models of intra‐guild predation

**DOI:** 10.1002/ece3.8375

**Published:** 2021-11-18

**Authors:** David H. Wise, Monica A. Farfan

**Affiliations:** ^1^ University of Illinois Chicago Illinois USA; ^2^ Colorado State University Ft. Collins Colorado USA

**Keywords:** field experiment, fungivore, generalist predators, IGP, intra‐guild predation, litter, mathematical models, micro‐arthropod, soil

## Abstract

We compared the response to resource enhancement of a simple empirical model of intra‐guild predation (IGP) to the predictions of published, simple mathematical models of asymmetric IGP (a generalist IG Predator that feeds both on a specialist IG Prey and a Resource that it shares with the IG Prey). The empirical model was a food‐web module created by pooling species abundances across many families in a speciose community of soil micro‐arthropods into three categories: IG Predator (large predatory mites), IG Prey (small predatory mites), and a shared Resource (fungivorous mites and springtails). By pooling abundances of species belonging to broadly defined functional groups, we tested the hypothesis that IGP is a dominant organizing principle in this community. Simple mathematical models of asymmetric IGP predict that increased input of nutrients and energy to the shared Resource will increase the equilibrium density of Resource and IG Predator, but will decrease that of IG Prey. In a field experiment, we observed how the three categories of the empirical model responded to two rates of addition of artificial detritus, which enhanced the food of fungivores, the Resource of the IGP module. By the experiment's end, fungivore densities had increased ~1.5× (ratio of pooled fungivore densities in the higher‐input treatment to plots with no addition of detritus), and densities of IG Predators had increased ~4×. Contrary to the prediction of mathematical models, IG Prey had not decreased, but instead had increased ~1.5×. We discuss possible reasons for the failure of the empirical model to agree with IGP theory. We then explore analogies between the behavior of the empirical model and another mathematical model of trophic interactions as one way to gain insights into the trophic connections in this community. We also propose one way forward for reporting comparisons of simple empirical and mathematical models.

## Graphical abstract

We compared the response to increased input of detrital resources of a simplified trophic grouping of many species of soil micro‐arthropods—the empirical model—to the behavior of published simple mathematical models of intra‐guild predation (IGP). IGP theory did not predict the overall behavior of the empirical model. We discuss how the failure of two simple models to agree could lead to a deeper understanding of the trophic connections in this speciose community.

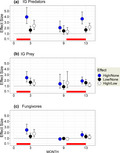

## 1 INTRODUCTION

Ecologists have long debated how simple mathematical models should be and how much to simplify complex food webs by lumping taxa into functional groups. For both modelers and empiricists, the *food chain* is the ultimate simplification. Consumers that feed extensively on more than one trophic level present a challenge to this simplification; ecologists have retained the abstract concept of trophic chains by labelling this feeding pattern *trophic*‐*level omnivory* (Pimm & Lawton, 1978). Consequences of this abstraction have proven profound, engendering a plethora of theoretical investigations into the influence on food‐web stability of trophic‐level omnivory and empirical research on its frequency of occurrence in nature.

**FIGURE 1:**
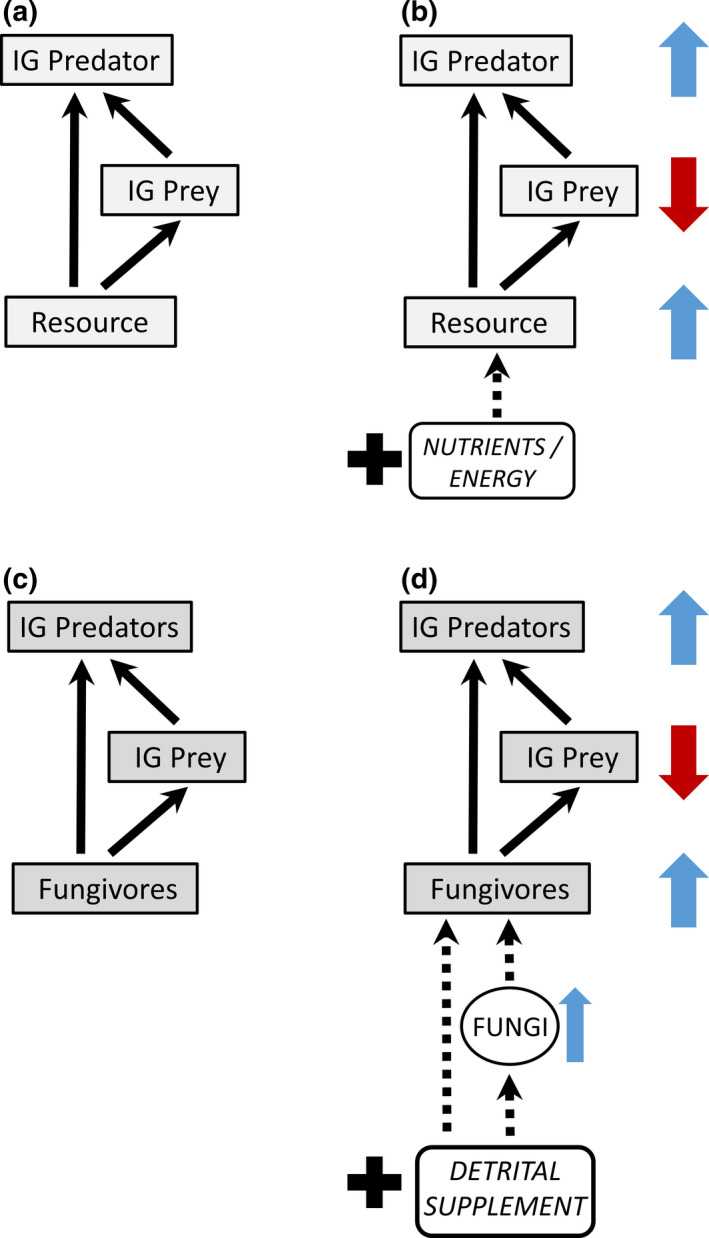
(a) Feeding relationships in asymmetrical intra‐guild predation (IGP). Arrows indicate direction of energy flow. (b) Summary of the behavior of early simple mathematical models of IGP (Diehl & Feissel, 2000; Holt & Polis, 1997) when productivity of the Resource increases above levels that were maintaining the coexistence of all compartments at equilibrium. Dashed arrow indicates an increase in exogenous sources of nutrients and/or energy that leads to increased Resource productivity. Colored arrows indicate model‐predicted direction of change in equilibrium density. We refer to this pattern as the behavior of *simple mathematical models* of IGP. (c) The *empirical model* of IGP for the micro‐arthropod community of the forest floor in which numerous taxa have been pooled into three model compartments. (d) Behavior of the *empirical model* of IGP in response to consistent input of a detrital supplement if the model's behavior follows predictions of *simple mathematical models* of IGP

An elegant and structurally simple example of trophic‐level omnivory is *intra*‐*guild predation* (*IGP*): the direct and indirect interactions between two predators (at least one of which feeds on the other) and a shared non‐predaceous prey. This *module* (Holt, 1997) is widespread in natural food webs (Arim & Marquet, 2004; Polis et al., 1989). The simplest version is *asymmetrical IGP* involving a generalist top *IG Predator*, a specialist intermediate *IG Prey*, and a shared *Resource* (Figure 1a). In nature, most IG Prey likely are generalists, not specialists; nevertheless, the asymmetrical abstraction is useful because predation on IG Prey by the IG Predator can often be considered the dominant interaction.

Early mathematical models of IGP are systems of three ordinary differential equations (ODEs) that describe the dynamics of the three IGP elements in terms of consumer–resource interactions, with the condition that the Resource grows to its carrying capacity in the absence of both consumers (Diehl & Feissel, 2000; Holt & Polis, 1997). Behaviors of these simple models can be complex, including unstable dynamics, alternate stable states, and narrow conditions for coexistence of Resource, IG Prey, and IG Predator. Nevertheless, we refer to them as *simple mathematical models* to distinguish them from later models that incorporate more realistic natural‐history features of the IGP module by expanding upon the simple system of three ODEs. Such later models incorporate biological realities such as age structure, ontogenetic changes in feeding behavior, cannibalism, and interactions with species outside the IGP module (Abrams, 2011; Hin & de Roos, 2019; Hin et al., 2011; Holt & Huxel, 2007; Mylius et al., 2001; Rudolf, 2007; Toscano et al., 2017).

In *simple mathematical models* of IGP, productivity of the shared Resource determines the number of IGP actors that can coexist (Diehl & Feissel, 2000; Holt & Polis, 1997). Only the Resource persists at very low productivities. As Resource productivity increases, only IG Prey and Resource can coexist, and at very high Resource productivity, only the IG Predator persists with the shared Resource. Only at intermediate levels of productivity is it possible for Resource, IG Prey, and IG Predator to coexist. Holt and Polis (1997) interpret this pattern to mean that for coexistence of all three, the IG Prey must be superior in exploitative competition for the Resource, and the IG Predator “should gain significantly” from consuming the IG Prey. The actual dynamics and patterns of invasibility differ between models, but a general pattern emerges. If one were to start with an equilibrium IGP system of three coexisting components, increasing productivity of the shared Resource should cause the density of IG Prey to decrease and densities of Resource and IG Predator to increase. This response to enhanced productivity of the shared basal resource we designate as the behavior of *simple mathematical models* (Figure 1b). According to these models, the system will eventually reach a new equilibrium that lacks IG Prey if Resource productivity reaches sufficiently high levels.

IGP modules are ubiquitous within food webs, yet it is a challenge to establish if the IGP model framework can successfully explain the dynamics of numerous generalist predators along shared resource gradients within a large food web. Researchers have compared predictions of mathematical models with the responses of laboratory microcosm models of IGP to enhanced resource productivity (Diehl & Feissel, 2000, 2001; Morin, 1999). In contrast, most, if not all, field experiments that have explicitly focused on the IGP module have manipulated predators, not the shared resource (Pahl et al., 2020). The more common approach is to compare model predictions with observed behaviors of IGP modules embedded in observed, but not intentionally manipulated, real‐world food webs (Pahl et al., 2020; Polis & Holt, 1992; Rudolf, 2007). These approaches, although valid ways to test the utility of theory, have serious limitations: (a) it is difficult to generalize congruence between microcosm and mathematical models to complex food webs in nature; (b) non‐manipulated natural food webs can encompass numerous IGP modules, making it a logistical challenge to compare model fit with all of them; and (c) the latter approach may unavoidably emphasize examples of congruence while tending to neglect failures of empirical and mathematical models to match (confirmation bias). Different approaches might overcome these drawbacks. We propose one such alternative.

Our argument is straightforward. If IGP is widespread throughout a food web, one would expect some of the numerous IGP modules to display similar synchronous dynamics, with others exhibiting out‐of‐phase dynamics. Exhaustively comparing the dynamics of these modules with different mathematical models of IGP interactions is not feasible, and as argued earlier, selecting a convenient few to compare has its drawbacks. However, if the dominant IGP modules exhibit synchronous dynamics, the sum of their behaviors should lead to an overall pattern across the food web that agrees with the predictions of simple mathematical models of IGP *if IGP is a dominant organizing principle*. Therefore, we selected a speciose natural food web that could reasonably be abstracted into a single, three‐compartment IGP module to create an *empirical model* of IGP. Our IGP model was created by summing the abundances of numerous taxa that we categorized, based upon their natural‐history attributes, as either IG Predator, IG Prey, or shared Resource. This IGP model/module could be viewed as a compartment embedded within a much larger web, but the pattern of connections to the larger web was not our focus.

We chose the species‐rich community of micro‐arthropods of the soil food web to construct our *empirical model*. Several features of this community prompted our choice: (a) micro‐arthropods such as mites and springtails are abundant in leaf litter and underlying soil horizons (Hopkin, 1997; Walter & Proctor, 2013); (b) soil micro‐arthropods are generalists that populate several trophic levels (Digel et al., 2014) yet are relatively similar in size and body plan (Bardgett, 2005), increasing the possibility of their populations exhibiting synchronous dynamics; (c) IGP appears to be more common in soil food webs than in many other webs (Digel et al., 2014); (d) numerous interactions occur within a square meter of the forest floor, making it feasible to establish many replicates of ecologically intact experimental units; and (e) it had already proven feasible to directly increase the rate of input of nutrients and energy to the shared Resource of our empirical model of IGP. The most‐abundant micro‐arthropods in our study community (mites and springtails) were classified into one of three categories: IG Predators, IG Prey, and Fungivores (the shared Resource) (Figure 1c). We then compared the response of the *empirical model* to increased input of nutrients and energy to the shared basal Resource with the behavior of *simple mathematical models* subjected to a similar bottom‐up perturbation (Figure 1d).

## 2 METHODS

### 2.1 Experimental design

Experimental units were replicated, fenced areas of the forest floor that received one of three levels of a detrital supplement. Replicates of comparable open, undisturbed areas served as a non‐experimental reference condition. In June 2014, 200 circular 1‐m^2^ plots were located within a 1‐ha area of forest at the Morton Arboretum in Lisle, Illinois, USA. One hundred and fifty plots, selected at random, were fenced with low aluminum flashing; the remaining 50 plots were left open as the reference (REF) treatment. Although there were no obvious gradients across the study site, plots were distributed among five blocks to account for possible effects of undetected gradients in soil properties. Three levels of detrital supplementation (None [0X], Low [1X], or High [4X]) were randomly assigned to the fenced plots. The added detritus was flakes of fruitfly‐culture medium mixed with chopped mushrooms and potatoes. The amount added per square meter every 2 weeks in the Low (1X) treatment was similar to that employed in an earlier experiment with unfenced plots in which densities of mites (Acarina) and springtails (Collembola) had increased after ~4 weeks (Chen & Wise, 1997, 1999). This rate of addition of the same detrital supplement to fenced and open plots in a later experiment increased fungal densities 2–3× as measured by ergosterol concentrations in the upper and lower leaf‐litter layers (Lawrence & Wise, 2017). Detrital enhancement was applied every 2 weeks from mid‐July through late September in 2014 (six applications) and from mid‐April through mid‐August in 2015 (nine applications).

Soil micro‐arthropods were sampled a week before detrital additions started (initial conditions: early July 2014) and 3 months later. Animals were sampled again in the fenced plots in early April the following year, just prior to resumption of detrital supplementation in the Low and High treatments, and were sampled in all plots at the end of the experiment in mid‐August 2015 (13 months after detrital supplementation commenced). Separate samples of leaf litter and the underlying organic soil horizon were taken to the laboratory where animals were extracted into 70% ethanol with a modified Berlese/Tullgren funnel (Henderson & Southwood, 2016). The leaf litter and underlying organic horizon are physically different, but many micro‐arthropods readily move between the two soil horizons, and discerning a clear boundary is often difficult. We therefore considered the organisms in these two layers to form a single community, and numbers in the two samples from each plot were combined for statistical modeling.

Further details on the research site, construction and arrangement of the experimental units, composition of the detrital subsidy, and micro‐arthropod sampling are given in Appendix S1.

### 2.2 IGP module

All adult micro‐arthropods were identified to at least the family level. Analysis of the community was restricted to mites (Subclass Acarina) and springtails (Order Collembola), the two most abundant taxonomic groupings of micro‐arthropods in our samples. Adults were classified as IG Predators (10 families), IG Prey (7 families), or Fungivores (23 families) based upon published field and laboratory studies of gut analyses or what individuals in each family have been directly observed to consume. Our category “Fungivores” includes organisms that not only graze fungal hyphae but also consume animal and plant detrital materials—hence their occasional designation as “microbi‐detritivores” (Scheu et al., 2003). For simplicity, we have designated them to be “Fungivores.” Juveniles of adult IG Predators of the Order Mesostigmata were classified as IG Prey because of their small size. Juvenile Fungivores of the Order Oribatida and Suborder Prostigmata could not be classified to family; they were categorized as Fungivores because practically all adult oribatids and prostigmatids in our samples were fungivores. Details appear in Appendix S2.

Categorization of diverse individuals into three broad IGP compartments is the heart of our research design. Clearly, this scheme oversimplifies, as the complexity of trophic interactions in soil is well documented. Nevertheless, despite the dietary generalism and plasticity of soil consumers, Collembola, Oribatida, and Prostigmata are primarily fungivorous, and the families identified as IG Prey and IG Predators behave primarily as predators (Appendix S2: Table S2).

### 2.3 Statistical modeling

The REF plots were not incorporated into analyses of detrital treatment effects because these areas of the forest floor differed from the experimental units in both fencing and detrital supplementation. The three experimental treatments were compared with the REF areas by visually inspecting the temporal patterns in densities. The REF treatment also was used in an explicit evaluation of a possible fence effect by comparing densities in REF and None treatments (which differed only in whether they were fenced) at the end of both field seasons.

**FIGURE 2:**
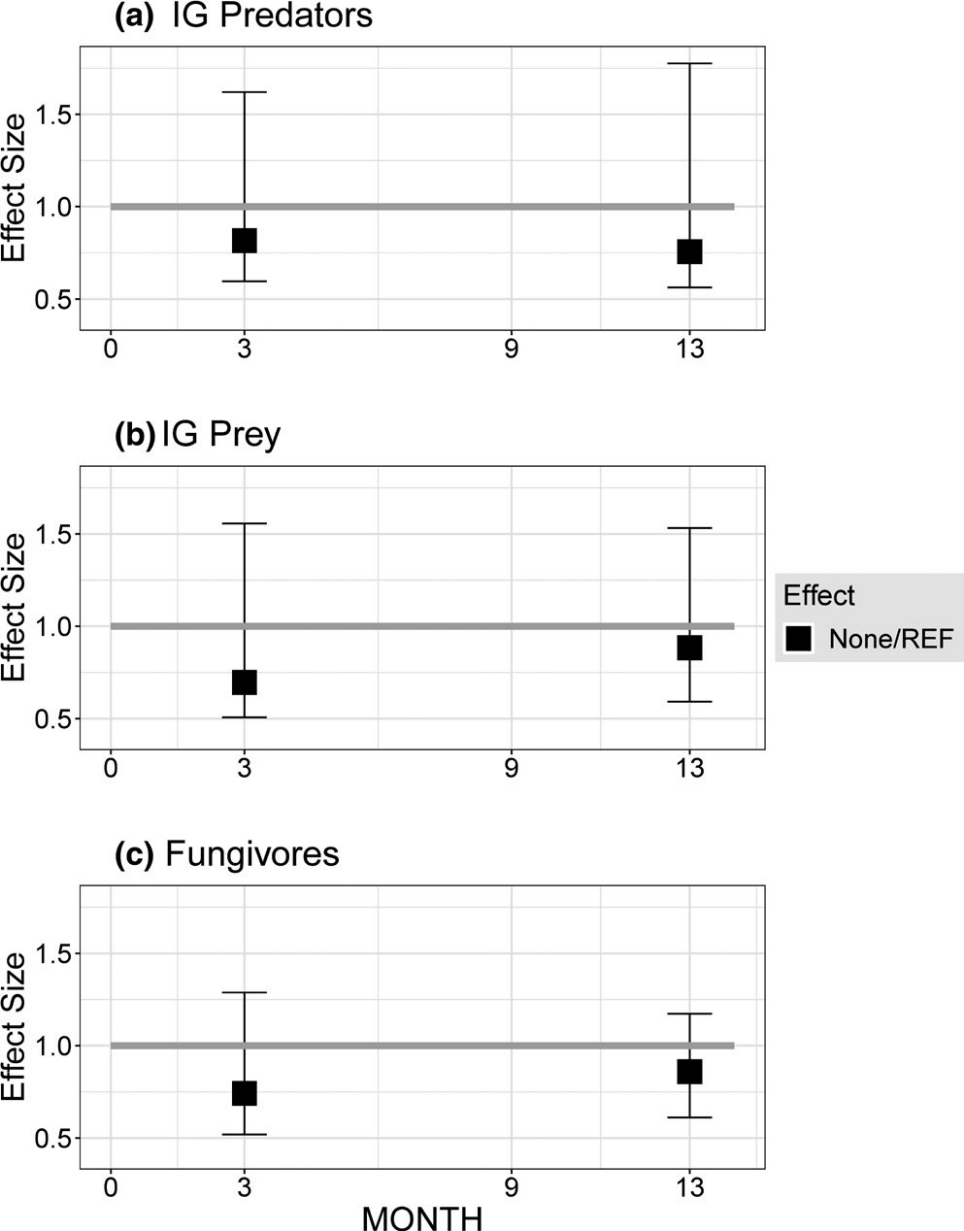
Effect of fencing on densities of components of the empirical IGP model in areas of the forest floor that did not receive a detrital supplement, expressed as the ratio of densities in the None (Fenced) treatment to densities in the REF (Not fenced) plots. Gray horizontal line represents absence of an effect (ratio = 1). MONTH = number of months since the beginning of the experiment, when the fencing was installed. No samples were taken in the REF plots at MONTH = 9

**FIGURE 3:**
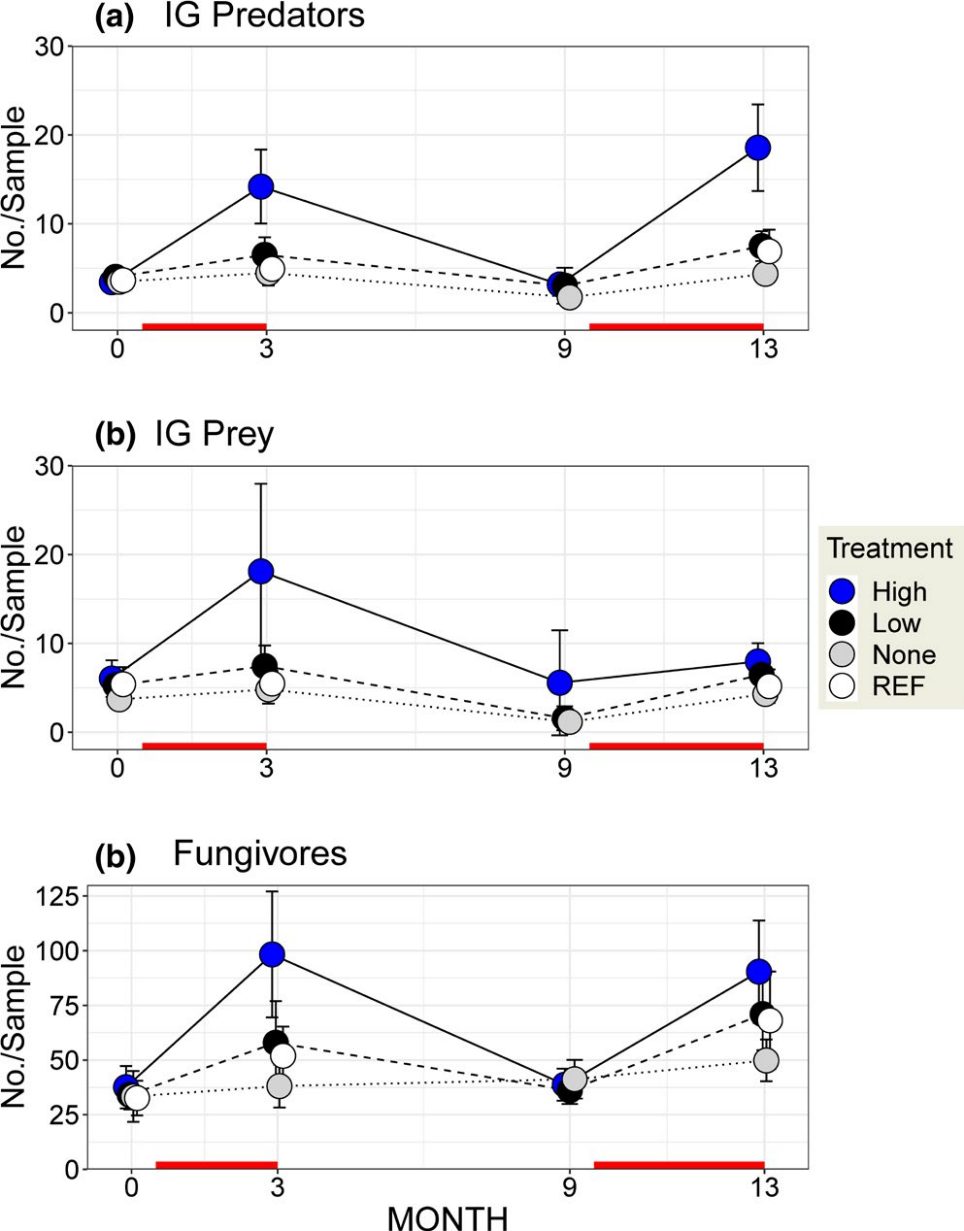
Mean abundances per sample with 95% CI's for the three compartments of the empirical IGP model in the three fenced detrital‐addition treatments (High (4X), Low (1X) or None (0X) rates of supplementation) and the unfenced reference area (REF). Red line along the abscissa represents the weeks when detritus was added. MONTH = number of months since the beginning of the experiment. Initial conditions (pre‐treatment abundances) are given at Month = 0. CI's smaller than the symbol are not given. No samples were taken in the REF plots at MONTH = 9

**FIGURE 4:**
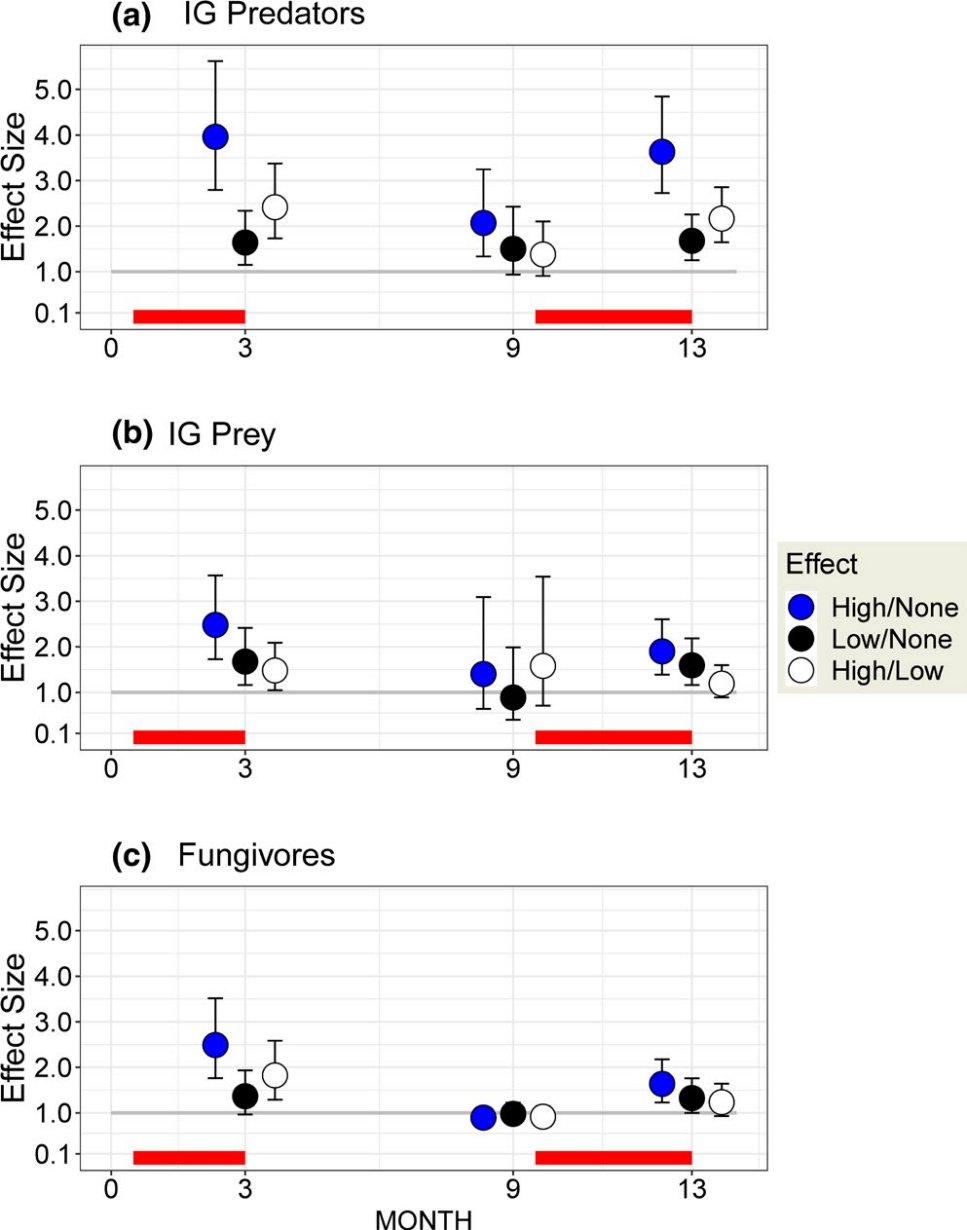
Model‐predicted effect size (response to detrital supplementation expressed as ratio of densities) with 95% CI's for the three compartments of the empirical IGP model. Red line along the abscissa represents the weeks when detritus was added. MONTH = number of months since the beginning of the experiment. CI's smaller than the symbol are not given. None = no supplementation (0X); Low = lower rate of supplementation (1X); High = four times the Low rate of supplementation (4X)

Evidence for a response to detrital supplementation in the three fenced treatments was the presence of a Treatment × Time interaction in a repeated‐measures, mixed‐effects linear or generalized linear model. Whether to retain Block as a factor, and the impact of extreme outliers on model behavior, was evaluated by whether ∆AIC > 2 and by testing the underlying mathematical assumptions of the simplified model. Because repeated‐measures models revealed treatment effects, differences between the None, Low, and High treatments were evaluated at each sampling period following the initial, pre‐treatment sample, with log(initial numbers + 1) as a covariate. Results are presented as model‐fitted effect sizes expressed as ratios (each detrital‐addition treatment compared with None, or a comparison of the two treatment levels (High/Low) with 95% confidence intervals (CIs). Details appear in Appendix S3. All statistical modeling, including generation of the plots in Figures 2, 3, 4, was done with R software (R Core Team, 2020).

## 3 RESULTS

We first present the results of model selection and simplification, followed by evidence that fencing did not adversely affect densities of micro‐arthropods within 1‐m^2^ areas of the forest floor, suggesting that our fenced plots enclosed a relatively intact, natural community. We then describe how densities responded to detrital addition in the fenced experimental units—the behavior of our empirical model.

### 3.1 Model selection and simplification

The initial models that adequately satisfied model assumptions were linear models with a log transformation for counts of Fungivores, and for counts of IG Prey and IG Predators, generalized linear models with a log link and the negative binomial family (Appendix S3.1). Block was retained as an additive, fixed effect in all models (Appendix S3.1). Removing extreme outliers improved all models for effects at individual sampling dates (Appendix S3.3).

### 3.2 Comparison of the None (0X) and Reference (REF) treatments

Fencing the plots had no discernable impact upon densities of any of the IGP categories in plots that had not received a detrital supplement (Figure 2, Appendix S3.4). Although densities in the None treatment (fenced, no supplementation) appear to have been about 25% lower than in the Reference plots (unfenced, no supplementation), confidence intervals for the mean effect size broadly overlapped an effect size = 1 (no effect). Of course, this comparison does not establish that fencing did not influence how detrital supplement affected the system; that question cannot be addressed directly with this experimental design. Nonetheless, this result suggests that fencing did not have a large impact on species interactions in the experimental units.

### 3.3 Change in densities in fenced, detrital‐addition plots (0X, 1X, and 4X)

Initial inspection of mean abundances per sample (Figure 3) reveals that densities of IG Predators, IG Prey, and Fungivores increased ~1.5–4× in response to the detrital treatment (*P*(Treatment × Time, Likelihood‐Ratio, *df* = 6) = 0.0001, 0.022, and 0.006, respectively; Appendix S3.1). The response appears to have been larger, and more consistent, for the High level of supplementation (Figure 3; and results of repeated‐measures modeling for Low and High treatments separately [Appendix S3.2]). Response to the High rate of supplementation may have been declining over the experiment for IG Prey (Figure 3b) but perhaps not for IG Predators and Fungivores (Figure 3a,c).

Does this pattern portend an eventual trajectory of densities of IG Prey in the High treatment becoming less than those in the None treatment, as predicted by simple mathematical models (Figure 1d)? Unfortunately, concluding congruence between simple empirical and mathematical models based solely upon these patterns is misleading for at least three reasons: (a) the broad CI on the mean density of IG Prey after 3 months makes interpretation of its temporal pattern problematic; (b) comparing densities in detrital‐addition plots with the contemporaneous control (None [0X]) is not a direct test of treatment effects; and (c) simply comparing mean densities of treatments ignores both the non‐normal distributions of many response variables and the reduction in error variance achieved by including block in the statistical model (Appendix S1). Statistical modeling of effect sizes over time is the more informative approach (Appendix S3.3).

### 3.4 Effect sizes over the course of the experiment

The temporal pattern of model‐fitted effects of the Low and High detrital enhancements leads to a different conclusion. The system's response was not congruent with the behavior predicted by simple mathematical IGP models when effect sizes are examined (Figure 4 vs. Figure 1b,d).

Effect size for the Low treatment (Low/None) for all IGP compartments at the end of each field season was ~1.5 or less (Figure 4, black circles, Months 3 and 13). After a hiatus in supplementation of 6 months (Month 9), Low‐treatment effects clearly had vanished for IG Prey and Fungivores (Figure 4b,c) but not for IG Predators (Figure 4a).

IG Predators displayed a consistently large response to High rates of supplementation, exhibiting densities ~4× higher than those in the None treatment at the end of both field seasons (Figure 4a, blue circles). A High‐treatment effect persisted for IG Predators after the 6‐month interruption in detrital addition but had dropped from ~4× to ~2× (Figure 4a, Month 9). Responses to the High treatment of both IG Prey and Fungivores were considerably weaker, declining from ~2.5 after the first season (Figure 4b,c; Month 3) to lower levels (~1.5×) at the end of the experiment (Figure 4b,c; Month 13). In contrast to the impact on IG Predators, the effects of the High treatment on densities of IG Prey and Fungivores had disappeared by Month 9 (Figure 4b,c).

One way to discern where the system may have been heading is to examine the overall pattern of concurrent changes in the High‐treatment effect (Figure 4, blue circles), High/Low comparison (Figure 4, white circles), and width of the error bars (95% CIs) on effect size. The response of IG Predators was the greatest and most consistent; that of Fungivores the weakest and most altered over time; and that of IG Prey intermediate but remarkably similar to that of the Fungivores in the final set of samples. The difference between IG Predators and Fungivores in their evolving responses to detrital supplementation over the experiment is striking (Figure 4a,c).

## 4 DISCUSSION

Deciding how trophic groups relate to modularity in the simplification of food‐web structure is a recurring theme in food‐web ecology (e.g., Gauzens et al., 2015). Our approach differs from most others. We started by defining the entire web as a single module and then used a field experiment to test the hypothesis that the module components would respond to a resource perturbation according to simple mathematical models of trophic interactions within the module, that is, according to IGP theory. Below, we evaluate the evidence for the validity of our hypothesis and the adequacy of our experimental design. We then conclude our discussion by comparing the results of our field experiment with a simple modeling approach that starts with an IGP configuration and then examines the equilibria of different trophic configurations along a resource gradient.

**FIGURE 5:**
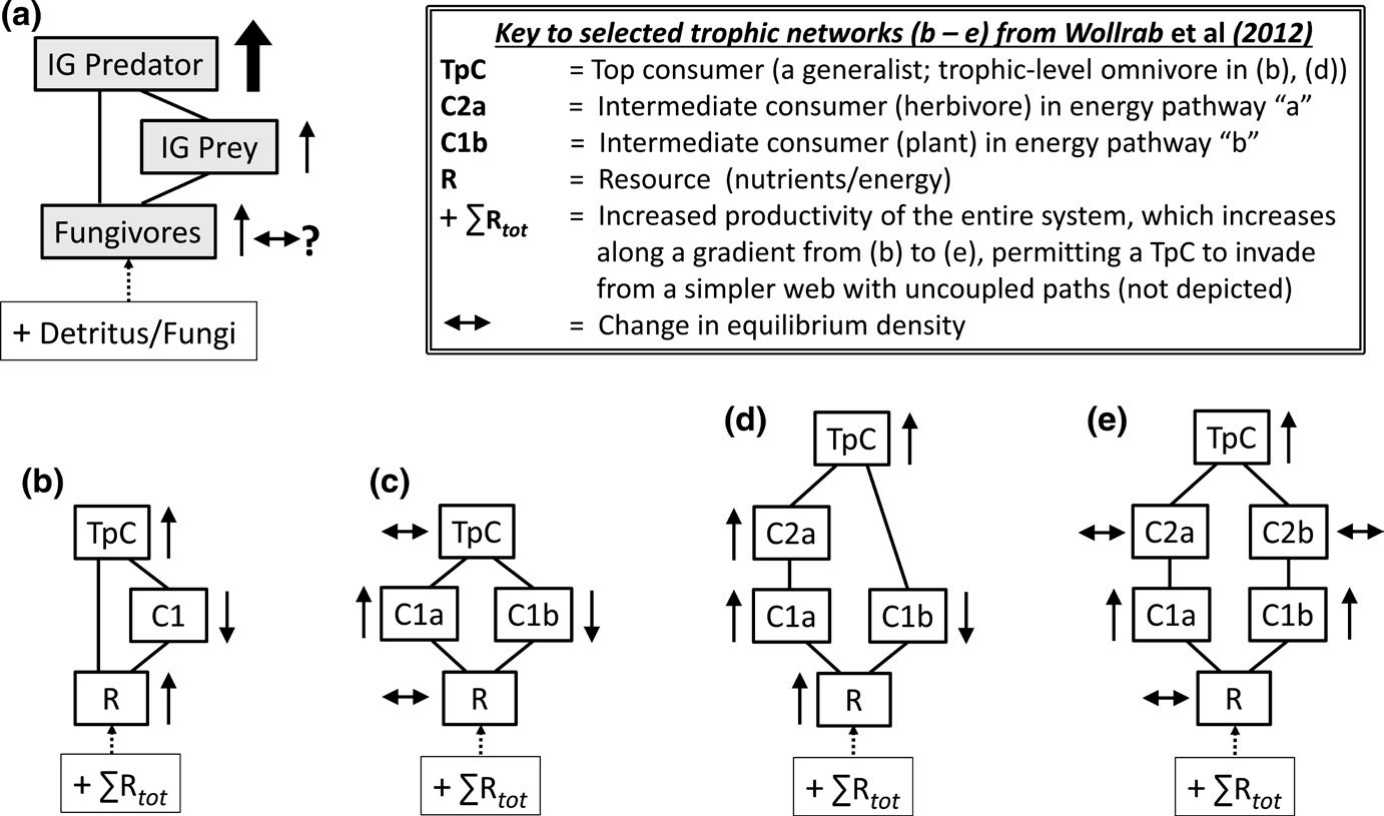
(a) The empirical IGP model's response to resource enhancement assuming that the temporal pattern of changing effect sizes in Figure 4 indicates eventual equilibrium states. Arrow direction indicates direction of change in equilibrium density, and differences in arrow width roughly reflect differences in effect size between compartments (whereas the arrows in (b)–(e) below only indicate direction of change in equilibrium density). The “?” next to the horizontal two‐headed arrow suggests there may be no change in equilibrium density for Fungivores, but that such a conclusion is equivocal. Lower diagrams (b)–(e) depict qualitative behaviors (changes in equilibrium densities) of four model food webs from (Wollrab et al., 2012). We have changed their coding of compartment names for generality. (b) Simple asymmetrical IGP (analogous structure to mathematical models depicted in Figure 1a, and the empirical model in (a) above), with TpC ~ IG Predator, C1 (plant) ~ IG Prey, and R (nutrient/energy) ~ Resource/Fungivores. The models of Wollrab et al. (2012) differ from the simple IGP models depicted in Figure 1a in the form of the equation describing the growth of “R” (explained in the text); the fact that equivalent compartments are interpreted to be dramatically different is less important. (c) Another intermediate consumer has been added to the food web and the IGP pattern disappears as the top consumer (TpC) now feeds on only one trophic level. (d) Addition of a second trophic level for intermediate‐level consumers, and reappearance of trophic‐level omnivory, but no IGP because energy channels “a” and “b” are separate. (e) Yet another second‐level intermediate consumer is added, removing trophic‐level omnivory

“It may be even more interesting when the predictions of a particular module model fail” (Holt, 1997). Classic, simple mathematical models of IGP failed to predict the overall behavior of our empirical model (Figure 5a compared with Figure 1d). Densities of IG Predators consistently increased four‐fold, providing confirmation of IGP theory for the top trophic level. Densities of fungivores increased initially, but this increase diminished as the experiment progressed, contrary to theory. The most striking failure of congruence between simple mathematical models and our empirical model is the failure of the IG Prey compartment to exhibit any hint of a decreased equilibrium density. Eventual extinction of IG Prey with increased resource input to the system is a major prediction of how a simple IGP module should behave. Hence, simple mathematical models of IGP failed to predict the overall behavior of the empirical IGP model.

Here, we should emphasize that we are restricting our discussion to the broad, qualitative behaviors of both models. For example, we are ignoring details of how parameter values of even simple mathematical models of IGP can affect dynamics and lead to alternate stable states. Model behavior becomes even more variable and complicated, as realistic details of biology are incorporated into the ODEs of simple IGP models. Throughout, we have emphasized relative magnitudes of responses by compartments of the empirical model as surrogates for strength of evidence for the qualitative pattern of how resource enhancement should alter equilibrium densities, and eventually coexistence, of all three trophic categories in the IGP module.

Was it the mathematical models, or the empirical model, that failed? We will examine some properties and weaknesses of both and will conclude that trying to identify one or the other type of model as the primary cause of non‐congruence may be short‐sighted and, in the long run, counter‐productive to building theory. Below, we explore some possible reasons for why the empirical IGP model failed to match the predictions of simple mathematical models and why this failure could be a step towards uncovering insights into how dominant trophic connections of the micro‐arthropod compartment of the larger soil food web might be structured.

### 4.1 System had not reached its new equilibrium state

This possibility, true for any manipulative field experiment, does not invalidate inferences from research in which several generations of the constituent species have elapsed. Our experimental model displayed a rapid response by the top trophic level that persisted for a second field season; the other two trophic groups also responded rapidly. Responses of the latter two appeared to be diminishing by the second field season, but the seasonal patterns were not strikingly different. This system likely had not yet reached its new equilibrium state, but even if convergence of experimental and baseline control treatments were to continue for many generations—proof of equilibrium—changes in extraneous driving variables might some day shift the system to an alternative stable state that might differ markedly from the former equilibrium. This is a conjecture, of course, but the true equilibrium state is ever‐elusive. What is germane is whether the system's behavior was sufficiently consistent to make generalizations about a match with what simple mathematical models predict. To achieve that goal, the pattern of response was sufficiently consistent.

### 4.2 Adding artificial detritus was an unrealistic perturbation

A cynic might conclude that our field experiment only established that densities of soil and litter micro‐arthropods were sliced‐potato limited. We would mildly retort that although the perturbation was itself not natural, it did encourage growth of the fungivores' natural food. Our detrital supplement can more than double densities of fungal hyphae in the leaf litter (Lawrence & Wise, 2017). Still, the addition was artificial. Our goal, however, was not to mimic natural variation in rates of supply of nutrients and energy to the soil food web. Instead, we aimed to uncover insights into the trophic structure of a natural community by perturbing the resource base in a non‐obtrusive manner. We enclosed undisturbed areas of the forest floor to reduce immigration and emigration, thereby increasing the likelihood that the system's response would reflect natural interactions in an intact subsystem of the forest. The magnitude of responses by module components (~1.5 to ~4×) was well within limits of natural spatial and temporal variation in densities of soil micro‐arthropods. The naturalness of the range of response supports the interpretation that the imposed resource perturbation uncovered natural processes.

### 4.3 Empirical model is not a valid IGP module

Our empirical model combines many more species than has been done previously for comparing model predictions with patterns of IGP in real‐world food webs (Pahl et al., 2020). Most empirical IGP models contain three species or three compartments that consist of taxonomically closely related species. Holt (1997) limits his definition of a food‐web “module” to strong interactions between three to six species, but he does point out that the term “module” sometimes refers to one of many “discrete blocks and interactions within more complex webs.” We have elected to use the broader definition, in which our “IGP module” refers to a food‐web compartment consisting of three clear functional groups. Defining functional groupings in trophic networks by combining numerous taxa is a widespread, fruitful research strategy (Greenop et al., 2018; McCary & Schmitz, 2021). The IGP module that we have defined is one of many compartments within an extensive soil food web that includes other arthropods, invertebrates such as nematode and annelid worms, vertebrates, and micro‐organisms. Thus, our empirical model, despite its complexity, qualifies as a “module” within the broader context of a food‐web compartment.

Nevertheless, our IGP module is itself a complex food web, a property that is fundamental to our goal of testing the hypothesis that *IGP is a dominant organizing principle* in a speciose community of generalist IGP predators and shared prey, such as that of soil micro‐arthropods. Thus, whether our abstraction of numerous families and species into an IGP module follows historical precedent of how IGP has been studied is only tangentially relevant. Our larger goal was not to test the applicability of IGP to simple networks of few species but to determine whether an unconventional application of the IGP theoretical framework might help advance our understanding of the structure and dynamics of trophic interactions in a species‐rich system.

### 4.4 The simple mathematical models of IGP we selected for comparison are too simple

The simple mathematical models we selected (Figure 1b; Diehl & Feissel, 2000; Holt & Polis, 1997) ignore complexities that have been incorporated into numerous more‐realistic mathematical models (e.g., age/size structure, ontogenetic changes in feeding behaviors, cannibalism, connections with species outside the module, and refuges for IG Prey) (Abrams, 2011; Hin & de Roos, 2019; Hin et al., 2011; Holt & Huxel, 2007; Mylius et al., 2001; Pahl et al., 2020; Rudolf, 2007; Toscano et al., 2017). Nevertheless, more complex and hence more “realistic” models of IGP can yield conclusions about how resource productivity affects coexistence that are similar to those of the classic simple models (Mylius et al., 2001; Pahl et al., 2020). It is also true, however, that adding complexities, such as refuges for IG Prey or strong connections to predators outside the IGP module, can increase the range of parameters over which coexistence of Resource, IG Prey, and IG Predator is possible (Pahl et al., 2020). Thus, it might be possible to conserve the basic IGP framework of our empirical model by discovering such features in the soil food web. This is a feasible and possibly productive direction for future research on this system. At this stage, though, we prefer to take a different route.

We will start by retaining our empirical model's trophic groups and will then compare their responses to resource enhancement to the predictions of a model of trophic organization that includes the IGP configuration but then examines the behavior of more‐complex trophic structures along a productivity gradient (Wollrab et al., 2012). The behavior of the IGP configuration in their model parallels that of simple models of IGP (Figure 1b), though their model is not entirely parallel in terms of the structure of the equations describing dynamics of the basal resource and consumers.

### 4.5 Exploring alternative trophic structures

Coalescing species of generalist micro‐arthropod predators and their non‐predaceous prey into the three compartments of the IGP module revealed that IGP was not the dominant organizing principle in this community, even though smaller subsets of species in this speciose community might follow predictions of mathematical models of IGP. Another trophic structure might be more congruent with the results of our field experiment. Below, we compare our findings with the behavior of a simple mathematical model of two alternate energy pathways (trophic chains of 2–5 levels) that are linked by a top generalist predator (Wollrab et al., 2012). We will focus on the responses of a subset of configurations of their model to increased productivity of the shared basal resource.

A caveat must be mentioned before we proceed. An anonymous reviewer pointed out that because the model of Wollrab et al. (2012) and IGP models differ in the dynamics of the basal resource, the validity of our comparison is questionable. The growth of fungivores in our real‐world empirical model would be limited by over‐consumption of fungal hyphae in the absence of predation; the shared resource in simple models of IGP also behaves this way because its dynamics are modeled by an ODE with a self‐damping term (e.g. Diehl & Feissel, 2000; Holt & Polis, 1997). In contrast, the size of the basal resource compartment in the model of Wollrab et al. (2012) is described by a non‐differential equation in which growth is limited solely by consumption by the next trophic level and total system productivity. They explicitly describe the basal resource in their model to be a nutrient that limits the growth of plants, but the biomasses of all other compartments are also expressed in units of nutrients. This equivalence is possible because their model assumes “… constant nutrient‐to‐carbon stoichiometries for all species. Consequently, nutrient and energy (= carbon) flows up a food chain are proportional …” (Wollrab et al., 2012). Their model is a closed system; so, in their simulations, they increase productivity of the basal resource by increasing the total amount of nutrients in the system.

Despite this difference in the manner in which dynamics of the basal resource is modeled in the two approaches, we argue that a comparison between the response of our empirical IGP model to resource enhancement and the model of Wollrab et al. (2012) can still yield insights. Our motivation is the striking similarity in the effect of increased productivity upon equilibria in their “IGP module” and analogous shifts in equilibria of Resource, IG Prey, and IG Predators in simple mathematical models of IGP.

Their simplest food web with a generalist predator is a depiction of trophic‐level omnivory (Figure 5b). Wollrab et al. (2012) identify this configuration as an “intraguild predation web” and interpret its behavior in terms of “… the frequently predicted extinction of the intraguild prey with enrichment” (Diehl & Feissel, 2000; Mylius et al., 2001). Of the three models with slightly more complex trophic structures, the two with 3–4 trophic levels match the behavior of our empirical model if intermediate trophic levels in the model are collapsed (Figure 5a compared with Figure 5d,e). The first model (Figure 5d) is a mixture of a three‐ and four‐trophic level web, which could be collapsed into a three‐level food chain if one assumes (a) that the net change in densities of C1a and C1b summed together is zero and (b) that one lumps all the species in C1a, C1b, and C2a into one category—such as IG Prey in our empirical model. The food web diagrammed in Figure 5d lacks an explicitly embedded IGP module but incorporates trophic‐level omnivory. The second model (Figure 5e) consists of two parallel food chains. Its behavior would agree with the behavior of our empirical model if (a) the response of the fungivores compartment eventually disappears and if (b) the species in the two intermediate trophic levels were lumped into one trophic category—such as IG Prey in our empirical model.

The congruence between the behavior of our empirical model and the two food‐chain models from Wollrab et al. (2012) suggests that a refinement of our knowledge of the natural history of soil micro‐arthropods in our system might reveal an empirical model consistent with Figure 5d or Figure 5e. For example, are some predators of fungivores preyed upon primarily by predators that are the prey of top generalist predators in the system, and do these top predators ignore fungivores? One might also discover pathways more similar to Figure 5e. Gut analyses of micro‐arthropods using molecular, biochemical, and stable‐isotope tools (Eitzinger et al., 2013) in experimental laboratory microcosms, and larger‐scale field experiments, could reveal whether such sets of pathways constitute reasonable empirical models to replace our simplified empirical IGP module.

With such more‐detailed natural history information, one could also tweak the mathematical models depicted in Figure 5d,e. One approach would be to incorporate IGP. Two possibilities are that C2a also preys upon C1b or that TpC also preys upon R. What dynamics would such feeding relationships produce? Are they consistent with an analogous empirical model based upon available natural history data? In addition, one could compare the pattern of response of the compartments of our empirical model to predictions of other three‐ and four‐trophic level models without, or with, IGP (e.g., Mylius et al., 2001; Oksanen et al., 1981 and vastly many more studies). The number of possibilities is staggering.

### 4.6 Moving forward

Tweaking simple mathematical and empirical models, rather than abandoning simplicity altogether, is one of several modeling philosophies. How to proceed is a constant challenge. Holt (1997) has argued that “… community modules provide community ecologists with a research path that with any luck skirts both the Scylla of unrealistic simplicity, and the Charybdis of unmanageable complexity.” Without deconstructing this nautically inspired metaphor, one can still conclude that its message is by no means mythical nor solely metaphorical. Our research paths will veer too close to either danger if we lack an objective way to evaluate the safety of our modeling tack. Imagine our conclusion if the behavior of our empirical model had matched that of the classic IGP model. We would have marveled at the ability of two simple models to agree and would have concluded that the collapsed soil micro‐arthropod community could indeed be modeled as a single IGP module. We would have surmised, with caution of course, that both models were simply simple enough. But we failed to find agreement, which may not be surprising to many ecologists researching IGP, who might dismiss this failure as obviously predictable. Always lurking is the danger that failure of an outrageously simplified empirical model to agree with predictions of simple mathematical models will not be published. Unpublished failures of agreement could suck us into a whirlpool of confirmatory bias as we struggle to avoid the dangers of unrealistic simplicity or excessive complexity in either mathematical or empirical models.

Ways exist to navigate around this whirlpool. We suggest the following research tack as one solution. Before the empirical model is investigated, publish in some readily accessible form (e.g., preregistration at https://plos.org/open‐science/preregistration/ or https://onlinelibrary.wiley.com/page/journal/20457758/homepage/registeredreports.html) the hypothesized agreement between empirical model and mathematical model (or any theoretical model, not necessarily one based on ODEs)—and how this agreement will be tested statistically. Then, publish and interpret results of the empirical test of the model no matter what the discovered level of agreement.

We did not preregister our empirical model and research design, but we recommend such an approach for the future. Such a comprehensive program is one way to help the community of ecologists better evaluate when a modeling approach is too simple—or simply too complex.

## CONFLICT OF INTEREST

Authors declare no competing interest.

## AUTHOR CONTRIBUTIONS

**David H. Wise:** Conceptualization (lead); data curation (supporting); formal analysis (lead); funding acquisition (equal); investigation (supporting); methodology (equal); project administration (supporting); resources (equal); software (equal); supervision (equal); validation (equal); visualization (lead); writing‐original draft (lead); writing‐review and editing (Lead). **Monica A. Farfan:** Conceptualization (supporting); data curation (lead); formal analysis (supporting); funding acquisition (equal); investigation (lead); methodology (equal); project administration (lead); resources (equal); software (equal); supervision (equal); validation (equal); visualization (supporting); writing‐original draft (supporting); writing‐review and editing (supporting).

## Supporting information

Appendix S1‐S3

## Data Availability

Data for this article are available at Dryad dataset, https://doi.org/10.5061/dryad.5qfttdz6k.
