# Supplementary material for: Effects of enhanced productivity of resources shared by predators in a food‐web module: Comparing results of a field experiment to predictions of mathematical models of intra‐guild predation
Source: Ecol Evol. 2021 Nov 18;11(23):17417–27. doi: 10.1002/ece3.8375 (PMC8668814; doi:10.1002/ece3.8375)
Supplement: Supplementary file 1 — Appendix S1‐S3 [file ECE3-11-17417-s001.docx]

**SUPPORTING INFORMATION**

Wise, D. H. and M. A. Farfan. 2021. Effects of enhanced productivity of resources shared by predators in a food-web module: Comparing results of a field experiment to predictions of mathematical models of intra-guild predation. *Ecology and Evolution.*

**Appendix S1: Experimental design**

The site of the experiment was a mixed mesic temperate forest bordering the remains of a pin oak (*Quercus palustris*) plantation. Previous management practices included prescribed burning and removal of European buckthorn (*Rhamnus cathartica*), though many exotic invasive herbaceous plants, such as garlic mustard (*Alliaria petiolata*), existed at the site.

In June 2014, 200 circular 1-m^2^ plots were established over a 21-day period within ~1-ha. Plots were divided evenly into five 12.2 x 15.2-m rectangular blocks, and were installed in a stratified design with daily installations spread across blocks and the four treatment types. One hundred and fifty of the 200 plots were fenced with aluminum flashing 25.4 cm wide, painted brown on one side and white on the other. Fencing was carefully buried ~7.6 cm into the ground while leaving the inside of the plot undisturbed. Another 50 plots (REF) were marked on the perimeter with Presco^TM^ steel wire stake flags (Forestry Suppliers, Inc., Jackson, Mississippi) but left unfenced.

The detrital supplementation treatment was a mixture of white potatoes that were peeled and chopped using a manual culinary chopper, and white button mushrooms sliced and then coarsely chopped using a knife. Mushrooms and potatoes were prepared ahead of time, placed in bags (enough for one plot in each), and frozen at -4°C until the morning that they were to be added to the experimental plots. Dry *Drosophila* medium flakes (Carolina Biological Supply, Inc.), also part of the detrital supplement, were sprinkled on the plot immediately before the chopped mushrooms and potatoes were dispersed across the plot. Treatments were:

- None (0X) – nothing added
- Low (1X) – 100 g chopped mushroom (wet weight), 100 g chopped potato (wet weight), 10 g Drosophila medium (dry weight)
- High (4X) – 400 g chopped mushroom (wet weight), 400 g chopped potato (wet weight), 40 g Drosophila medium (dry weight)

Soil micro-arthropods were sampled 10-18 July 2014, prior to the beginning of detrital supplementation the first field season (initial conditions); 27 September - 25 October 2014; 11-18 April 2015, prior to the beginning of detrital supplementation the second field season; and 8-15 August 2015, the end of the experiment. Litter samples were taken for each plot using a “grab” technique, in which a sample the diameter of the collector’s hand was extended fully and all litter within that area “grabbed” with one hand and bagged immediately in a Ziploc^TM^ bag. Soil samples were collected from the center of the area where litter was just collected using a standard bulb planter (Home Depot) ~6 cm in diameter and ~7 cm long, which was inserted into the ground using a twisting motion and lifted out with the soil sample inside. Soil samples were immediately bagged in Ziploc^TM^ bags for transport back to the lab. Soil and litter samples were kept in coolers for transport to the laboratory and stored at 11°C until they could be extracted. Arthropods from litter and soil samples were extracted separately one to three days after collection in a modified Berlese / Tullgren funnel into 70% ethanol. Mites and Collembola were identified to family using a high-resolution dissecting microscope. Approximately 200 specimens of mites, and 200 of Collembola, were chosen for mounting on glass slides for further taxonomic identification. Both mites and Collembola were cleared of internal tissue using lactophenol or laboratory-grade specimen clearing fluid (BioQuip Products, Inc.) for 1 to 7 days depending on the level of sclerotization of the specimen. Specimens were then mounted on slides using PVA mounting medium (BioQuip Products, Inc.) and placed in an oven at 50°C for approximately one week.

More samples were collected over the two years than could be sorted and identified because of the time required to process samples. All 2014 samples were sorted and analyzed, but micro-arthropods were not sorted and identified in samples collected from the REF plots in April 2015 (Month = 9). Furthermore, a randomly selected subset of the remaining April 2015 samples (90/150; 30/50 for each experimental treatment) was analyzed. Micro-arthropods were identified in all 200 samples from the last sampling date, except for one sample that was lost.

**Appendix S2: IGP module**

All adult specimens were identified to at least the family level. Assignment to trophic category was based upon the published literature (“References” in the RH column of Table S2 below) reporting field and laboratory studies of what adult individuals in each family had been directly observed to consume, or to have consumed based upon gut analyses. Although most adult Mesostigmata were categorized as IG Predators, all juvenile mesostigmatids (which could not be identified to family) were categorized as IG Prey because of their small size. Adults of only two mesostigmatid families were also classified as IG Prey because of their size. Juveniles of Oribatida and Prostigmata, which also could not be identified to family, were classified as fungivores because practically all adult oribatids and prostigmatids in our samples were classified as fungivores. Table S2 gives the assignment to IGP categories of mites and Collembola according to the above criteria.

A total of 45,177 specimens (73.5% from the litter, 26.5% from the lower soil horizon) were placed into one of the three IGP trophic categories (36,594 Fungivores, 4,253 IG Prey, and 4,330 IG Predators).

**Table S2**

| **Family Order or References**  **Suborder** | | |
| --- | --- | --- |
| **IG Predators** |  |  |
| Laelapidae | Mesostigmata | Lindquist et al. 2009 |
| Macrochelidae | Mesostigmata | Koehler 1999 |
| Pachylaelapidae | Mesostigmata | Koehler 1999; Klarner et al. 2013 |
| Parasitidae | Mesostigmata | Koehler 1999 |
| Parholaspididae | Mesostigmata | Koehler 1999 |
| Podocinidae | Mesostigmata | Evans and Hyatt 1957 |
| Ologamasidae | Mesostigmata | Lee 1974 |
| Veigaiidae | Mesostigmata | Koehler 1999 |
| Rhagidiidae | Trombidiformes | Walter et al. 2009 |
| Trombidiidae | Trombidiformes | Walter et al. 2009 |
|  |  |  |
| **IG Prey** |  |  |
| Ascidae | Mesostigmata | Koehler 1999; Walter et al. 1988 |
| Rhodacaridae | Mesostigmata | Koehler 1999 |
| Bdellidae | Trombidiformes | Walter et al. 2009 |
| Cunaxidae | Trombidiformes | Walter et al. 1988 |
| Eupodidae | Trombidiformes | Walter et al. 2009 |
| Microtrombidiidae | Trombidiformes | Walter et al. 2009 |
| Pomerantziidae | Trombidiformes | Walter et al. 2009 |
| Juvenile Mesostigmata |  |  |
| **Fungivores** |  |  |
| Entomobryidae | Collembola | Potapov et al. 2016 |
| Isotomidae | Collembola | Booth and Anderson 1979;  Potapov et al. 2016 |
| Hypogastruridae | Collembola | Potapov et al. 2016 |
| Neelidae | Collembola | Christiansen et al. 2009 |
| Onychuridae | Collembola | Bengtsson and Rundgren 1983 |
| Sminthuridae | Collembola | Potapov et al. 2016 |
| Tomoceridae | Collembola | Potapov et al. 2016 |
| Aphelacaridae | Oribatida | Smith et al. 2011 |
| Carabodidae | Oribatida | Schneider and Maraun 2005;  Schneider et al. 2004 |
| Ceratozetidae | Oribatida | Mitchell and Parkinson 1976 |
| Galumnidae | Oribatida | Schneider et al. 2004 |
| Licnermaeidae | Oribatida | Norton and Behan-Pelletier 2009 |
| Oribatellidae | Oribatida | Schneider and Maraun 2005 |
| Oripodidae | Oribatida | Norton and Behan-Pelletier 2009 |
| Phthiracaridae | Oribatida | Schneider and Maraun 2005 |
| Scheloribatidae | Oribatida | Mitchell and Parkinson 1976; Schneider et al. 2004; Schneider et al. 2005 |
| Tectocepheiidae | Oribatida | Schneider et al. 2004 |
| Unduloribatidae | Oribatida | Pfingstl and Krisper 2010 |
| Microdispidae | Prostigmata | Walter et al. 2009 |
| Pygmephoridae | Prostigmata | Cross and Kaliszewski 1988; Zou et al. 1993 |
| Scutacaridae | Prostigmata | Kaliszewski et al. 1995 |
| Tarsonemidae | Prostigmata | Walter et al. 2009 |
| Tydeidae (*Tydeus* sp.)**  Juvenile Oribatida  Juvenile Prostigmata | Prostigmata | Baker 1965; Baker 1970 |

** These taxa have been observed to consume resources at different trophic levels, but litter/soil-dwelling species tend toward fungivory.

**References cited in Table S2**

Baker, E. W. (1965). A review of the genera of the family Tydeidae (Acarina). *Advances in* *Acarology*, 2, 95-133.

Baker, E. W. (1970). The genus *Tydeus*: Subgenera and species groups with descriptions of new species (Acarina: Tydeidae). *Annals of the Entomological Society of America*, 63, 163-177.

Bengtsson, G., and S. Rundgren. (1983). Respiration and growth of a fungus, *Mortierella isabellina*, in response to grazing by *Onychiurus armatus* (Collembola). *Soil Biology and Biochemistry*, 15, 469-473.

Booth, R. G., and J. M. Anderson. (1979). The influence of fungal food quality on the growth and fecundity of *Folsomia candida* (Collembola: Isotomidae). *Oecologia*, 38, 317-323.

Christiansen, K. A., P. Bellinger, F. Janssens, V. H. Resh, and R. T. Cardé. (2009). Collembola: (Springtails, Snow Fleas). Pages 206-210 *Encyclopedia of Insects*. Academic Press, San Diego.

Cross, E. A., and M. J. Kaliszewski. (1988). The life history of a mushroom pest mite, *Pediculaster flechtmanni* (Wicht) (Acari: Pygmephoroidea), with studies of alternate morph formation. *Environmental Entomology*, 17, 309-315.

Evans, G. O., and K. H. Hyatt. (1957). The genera *Podocinum* Berl. and *Podocinella* gen. nov.(Acarina: Mesostigmata). *Journal of Natural History*, 10, 913-932.

Kaliszewski, M., F. Athias-Binche, E. E. Lindquist, J. R. Baker, R. Muller, and D. Rollinson. (1995). Parasitism and parasitoidism in Tarsonemina (Acari: Heterostigmata) and evolutionary considerations. *Advances in Parasitology*, 35, 335-367.

Klarner, B., M. Maraun, and S. Scheu. (2013). Trophic diversity and niche partitioning in a species rich predator guild – Natural variations in stable isotope ratios (13C/12C, 15N/14N) of mesostigmatid mites (Acari, Mesostigmata) from Central European beech forests. *Soil Biology and Biochemistry*, 57, 327-333.

Koehler, H. H. (1999). Predatory mites (Gamasina, Mesostigmata). *Agriculture, Ecosystems & Environment*, 74, 395-410.

Lee, D. C. (1974). Rhodacaridae (Acari: Mesostigmata) from near Adelaide, Australia. 3. Behaviour and development. *Acarologia*, 16, 21-44.

Lindquist, E. E., G. W. Krantz, and D. E. Walter. (2009). Order Mesostigmata. Page 124 *in* G. W. Krantz and D. E. Walter, editors. *A Manual of Acarology*. Texas Tech University Press, Lubbock, Texas.

Mitchell, M. J., and D. Parkinson. (1976). Fungal feeding or Oribatid mites (Acari: Cryptostigmata) in an aspen woodland soil. *Ecology*, 57, 302-312.

Norton, R. A., and V. M. Behan-Pelletier. (2009). Suborder Oribatida. Page 430 *in* G. W. Krantz and D. E. Walter, editors. *A Manual of Acarology*. Texas Tech University Press, Lubbock, TX.

Pfingstl, T., and G. Krisper. (2010). Development and morphology of *Unduloribates undulatus* (Berlese, 1914)(Acari: Oribatida) and some remarks on the Unduloribatidae. *Acta Zoologica Academiae Scientiarum Hungaricae*, 56, 119-138.

Potapov, A. A., E. E. Semenina, A. Y. Korotkevich, N. A. Kuznetsova, and A. V. Tiunov. (2016). Connecting taxonomy and ecology: Trophic niches of collembolans as related to taxonomic identity and life forms. *Soil Biology and Biochemistry*, 101, 20-31.

Schneider, K., and M. Maraun. (2005). Feeding preferences among dark pigmented fungal taxa (“Dematiacea”) indicate limited trophic niche differentiation of oribatid mites (Oribatida, Acari). *Pedobiologia*, 49, 61-67.

Schneider, K., S. Migge, R. A. Norton, S. Scheu, R. Langel, A. Reineking, and M. Maraun. (2004). Trophic niche differentiation in soil microarthropods (Oribatida, Acari): evidence from stable isotope ratios (15N/14N). *Soil Biology and Biochemistry*, 36, 1769-1774.

Schneider, K., C. Renker, and M. Maraun. (2005). Oribatid mite (Acari, Oribatida) feeding on ectomycorrhizal fungi. *Mycorrhiza*, 16, 67-72.

Smith, I. M., E. E. Lindquist, and V. Behan-Pelletier. (2011). Mites (Acari) of the Montane Cordillera ecozone. Pages 193-268 *in* G. G. E. Scudder and I. M. Smith, editors. *Assessment of species diversity in the Montane Cordillera ecozone*. Royal British Columbia Museum, Victoria, B.C.

Walter, D. E., H. W. Hunt, and E. T. Elliot. (1988). Guilds or functional groups? an analysis of predatory arthropods from a shortgrass steppe soil. *Pedobiologia*, 31, 247-260.

Walter, D. E., E. E. Lindquist, J. M. Smith, D. R. Cook, and G. W. Krantz. (2009). Order Trombidiformes. Page 233 *in* G. W. Krantz and D. E. Walter, editors. *A Manual of Acarology*. Texas Tech University Press, Lubbock, TX.

Zou, P., J.-R. Gao, and E.-P. Ma. (1993). Preliminary studies on the biology of the pest mite *Luciaphorus auriculariae* (Acari: Pygmephoridae) infesting Jew's ear mushroom *Auricularia polytricha* in China. *Experimental and Applied Acarology* 17, 225-232.

**Appendix S3: Statistical modeling**

We first describe in detail the modeling of the ***Treatment x Sample(Time)* interaction**, since this initial modeling influenced the structure of all subseqent models. We then report results of ***Treatment x Sample* interactions for two treatment levels at a time** (High, None; and Low, None); modeling of **three *Treatment* effects** (High/None, Low/None, and High/Low) for each of the three sample dates post-initial conditions (S1, S2, S3); and then the modeling of the ***Fence*** effect (None/REF).

**S3.1 Treatment (detrital addition) x Time (sample) interaction as evidence of a Treatment effect.**

**Fungivores:**

Counts of Fungivores per experimental unit were high with few zeros. The slightly asymmetrical distribution of Fungivore counts was corrected by a log(x+1) transformation, making a mixed-effects Linear Model (LMM) the simplest model. Adequacy of assumptions for a LMM was confirmed by analyzing plots of standardized residuals against fitted values, Treatment, Time (Sample) and Block (plots appear below).

**IG Prey and IG Predators:**

Counts of IG Prey and IG Predators were much lower than those of Fungivores, and were heavily skewed with substantial numbers of zeros. Thus, a priori, a linear model was not appropriate to model these response variables. We first tried a Poisson generalized mixed-effects linear model (GLMM), but the over-dipersion index (OD) was always >> 1. A GLMM with the negative binomial family and a log link successfully modeled the data based upon (1) OD’s close to 1 (1.07 (*P*  = 0.12) for IG Prey; 0.89, (*P* = 0.96) for IG Predators); and (2) adequacy of assumptions for a LMM as confirmed by analyzing plots of Pearson residuals against fitted values, Treatment, Time (Sample) and Block (plots appear below).

**Block**

In all the above models, block was treated as a fixed, additive factor. Block was not treated as a random variable because there were only five levels of block. Dropping the effect of Block from the model increased AIC by 2.7 (*P* = 0.029) for Fungivores by 22.6 (*P* < 0.001) for IG Prey, and by 10.5 (*P*  = 0.001) for IG Predators.

Block was treated as an additive factor because any Treatment x Sample x Block interactions would have been very difficult to interpret, since no known environmental differences were associated with the “Block” factor; and in addtion, complex interactions are difficult to detect statistically. Most importantly, we wanted to use models that were as simple as possible to interpret, but that also minimized error variance (as was the case with Block as an additive factor). Neverthless, we did run full models with the three-way interactions, and found no evidence of a consistently, strong three-way interaction. For fungivores, the additive model had a much lower AIC than the full model (∆AIC = 42.5); and for IG Prey, ∆AIC = 0.3. Thus, for these two variables the simpler model was justified. For IG Predators, however, the full model had a lower AIC ((∆AIC = 10.9, *P* = 0.018 by drop1() test). However, of the 24 possible three-way interactions in the full GLMM (comparing Low and High treatments with None; and S1, S2, and S3 with S0), only one three-way interaction had a *P* < 0.10 (*P* = 0.08). Thus, the overall pattern for the response variables suggested that including the three-way interaction in the models was not justified.

**Evidence of a Treatment Effect**

Because initial, pre-treatment conditions were part of the model, value of the Treatment x Time interaction is evidence of an effect of Treatment. These are mixed-effect models because of the repeated-measures sampling design (in the R code, modeled as “**+ 1 | Plot**”). Overall effect of treatment was based upon the Log Ratio Test of the Treatment x Time interaction (**Treat:RmTot** in the R Code), which appears after plots of model assumptions

**Fungivores**


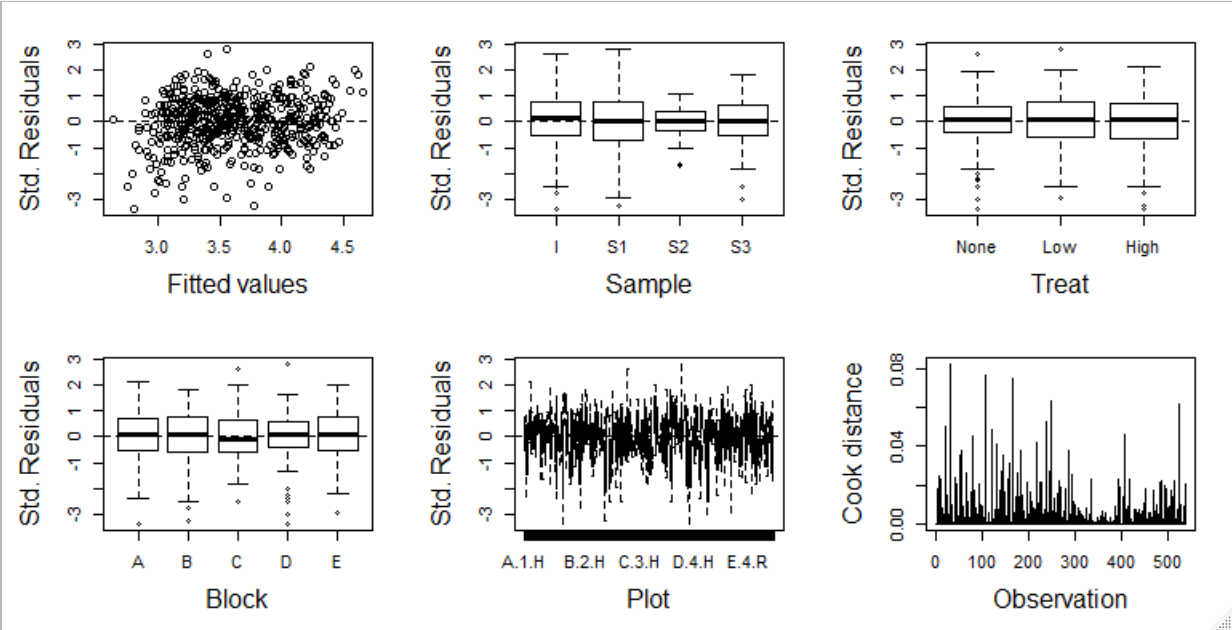


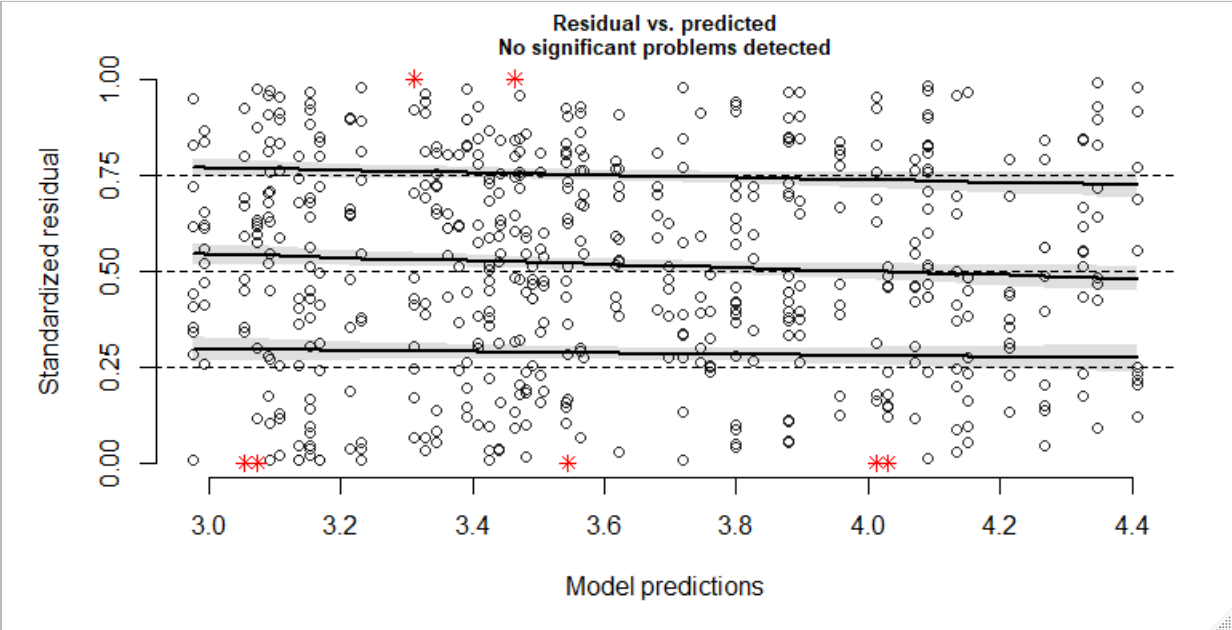


> drop1(Model, test = "Chisq")

log(Fungiv + 1) ~ Treat * RmTot + Block + (1 | Plot)

npar AIC LRT Pr(Chi)

<none> 1388.5

Block 4 1391.2 10.766 0.029327

**Treat:RmTot 6 1394.5 17.998 0.006237**

**IG Prey**


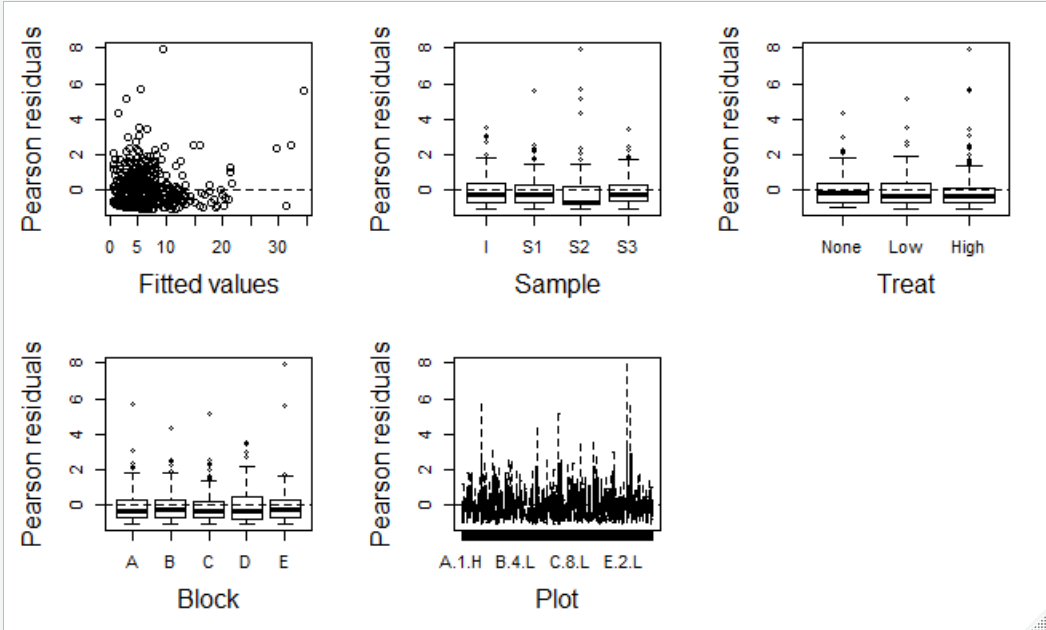


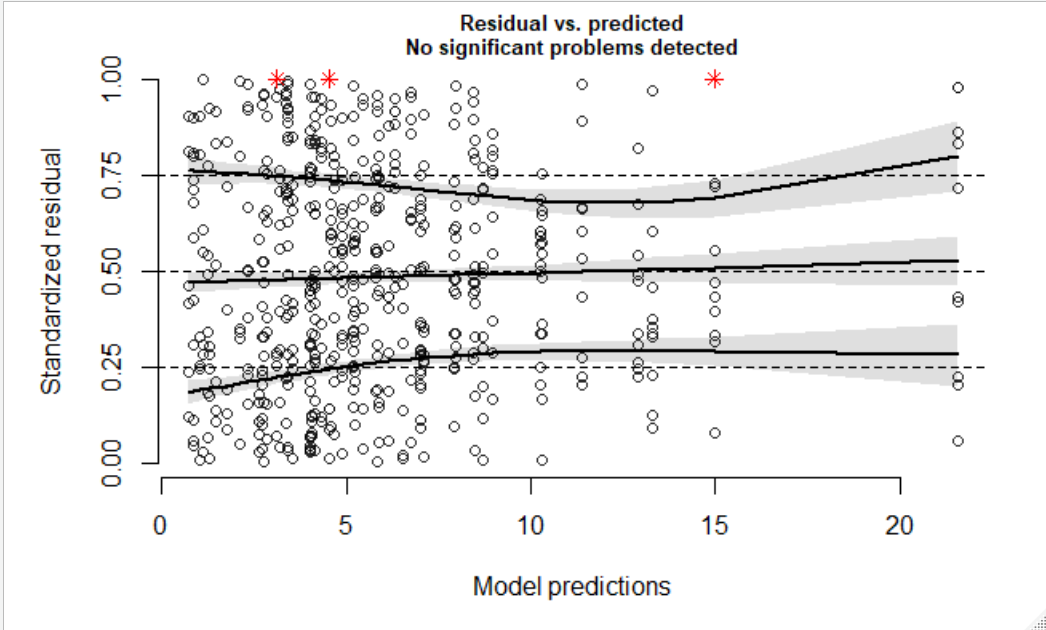


> drop1(Model, test = "Chi")

IGPrey ~ Treat * RmTot + Block + (1 | Plot)

Df AIC LRT Pr(>Chi)

<none> 2974.9

Block 4 2997.5 30.588 3.715e-06

**Treat:RmTot 6 2977.7 14.754 0.02226**

**IG Predators**


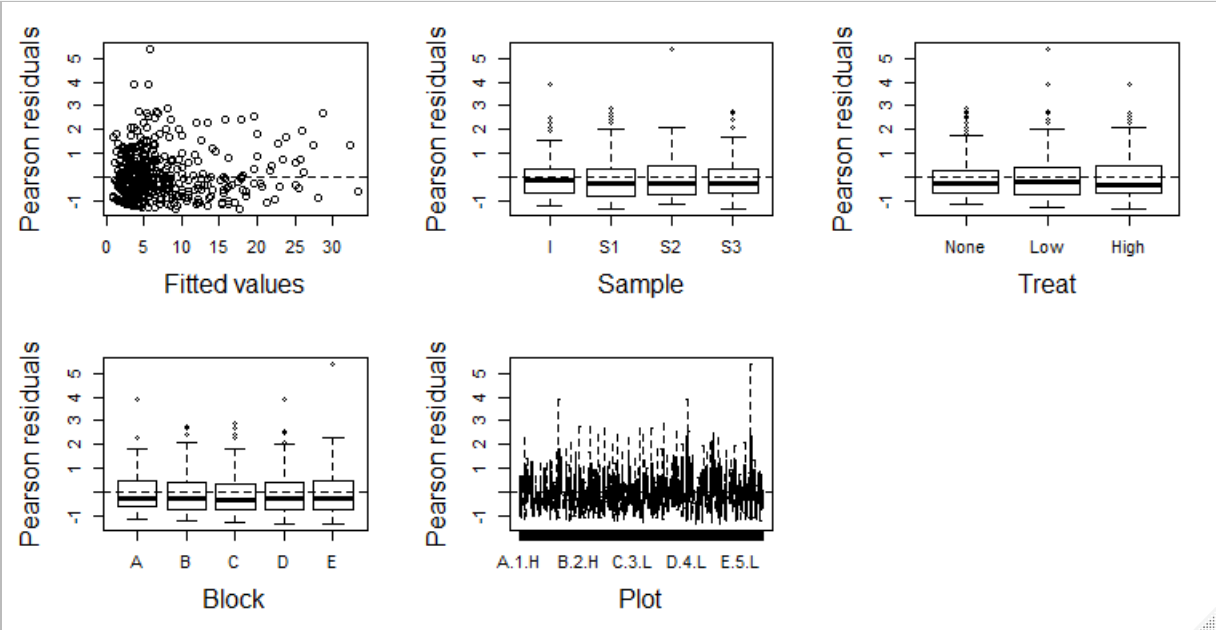


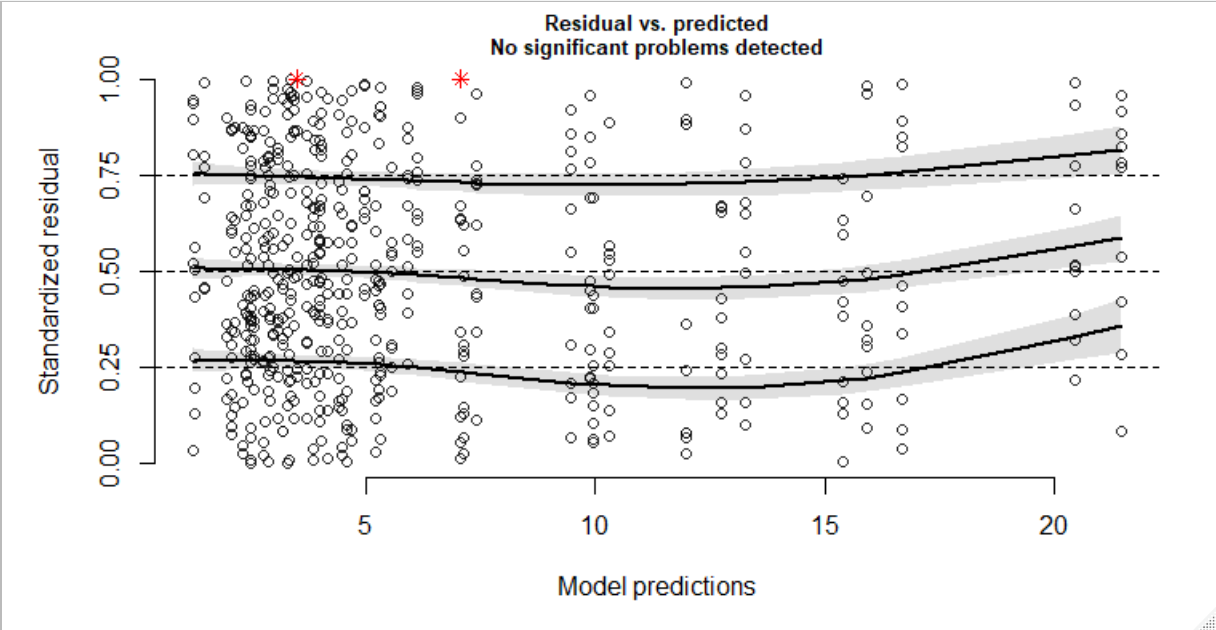


> drop1(Model, test = "Chi")

Predat ~ Treat * RmTot + Block + (1 | Plot)

Df AIC LRT Pr(>Chi)

<none> 2938.8

Block 4 2949.3 18.538 0.0009682

**Treat:RmTot 6 2968.7 41.900 1.925e-07**

**S3.2 Treatment (detrital addition) x Time (sample) for Low and High treatments modeled separately.**

The High treatment displayed the strongest evidence of a treatment effect for all three IGP categories. Results for models with only two treatment levels (High, None) are: *P*(Treatment x Time) < 0.0001 after both 3 and 13 months for IG Predators; *P*(Treatment x Time) = 0.007 and 0.019 after 3 and 9 months, respectively, for IG Prey; and *P*(Treatment x Time) = 0.0009 and 0.068 after 3 and 13 months, respectively, for Fungivores.

Evidence of a consistent response to the Low treatment is lacking for all categories. (*P*(Treatment x Sample) > 0.20 for all comparisons except for IG Predators after 13 months (*P* = 0.056).

**S3.3 Effect Sizes: Detrital supplementation**

Because of clear evidence of a Treatment x Time interaction (S3.1), we next determined the effect of detrital addition for each sampling date after detrital addition had commenced. Although the repeated-measures models uncovered clear evidence of an effect of the detrital supplement only for the High treatment for all IGP categories, effect sizes were also calculated for the Low treatment for comparison, and also to enable calculation of the ratio High/Low.

The model used to calculate effect sizes was parallel in structure to the repeated-measures models, except that Time(Sample) was no longer a factor, and we added log(Initial Counts + 1) to account for pre-Treatment differencs in densities, yielding the R code:

Response Variable ~ Treatment + Block + log(Initial Counts + 1),

where Treatment = three levels of the detrital Supplementation (None, Low and High), and Initial Counts = the numbers in the sample S0.

Unlike the situation for the mixed-effect models, which modeled the entire data set, extreme outliers often prevented model assumptions from being met, or in a few cases, prevented convergence of the GLM. These outliers were probably not mistakes in data processing, but simply reflected the highly heterogenious soil envirnment. This heterogeneity became more pronounced when subsets of the full data set were analyzed. Using points identified by the R program on plots of Cook’s distance as a starting point to identify possibly influential outliers, the degree to which the model with the full data set met assumptions was compared to that of a model with one-or-more outliers removed. A model with the three worst outliers removed from the data set (3/50 observations for S2 and S4, 3/30 observations for S2) was always a better fit, often dramatically so, as judged by ∆AIC; by an OD closer to 1; and better-behaved plots of residuals. Table S3.3.1 (following page) summarizes the results of this model selection. Effect sizes (High/None. Low/None and High/Low) and 95% CI’s were then obtained by modeling the data with the three most-extreme outliers removed.

**Table S3.3.1** Removing the three most-extreme outliers improved all models. All models with the outliers removed had lower AIC values, and in seven out of nine models, better-behaved residuals. All GLM’s with outliers removed had lower OD’s, all very close to 1 (*P*’s > 0.30)

**ALL = Model with all the data**

**RED = Model with 3 most-extreme outliers removed**

**OD = Index of Over-Dispersion**

**CD = Cook’s Distance**

| **Response**  **Variable** | **S** | **AIC** | | **∆AIC** | **OD** | | **Max**  **CD**  **for**  **RED** | **Plots of Residuals**  **Improvement / RED** |
| --- | --- | --- | --- | --- | --- | --- | --- | --- |
|  |  | **ALL** | **RED** |  | **ALL** | **RED** |  |  |
| **Fungivores** |  |  |  |  |  |  |  |  |
|  | S1 | 416 | 386 | 30 | N/A | N/A | .048 | Better / Excellent |
|  | S2 | 138 | 109 | 29 | N/A | N/A | .069 | Better / Excellent |
|  | S3 | 360 | 323 | 37 | N/A | N/A | .050 | Better / Excellent |
| **IG Prey** |  | | | | | | | |
|  | S1 | 935 | 874 | 61 | 1.42 | 1.06 | .105 | Better!! / Good |
|  | S2 | 337 | 279 | 58 | 1.20 | 1.02 | .180 | Better / Good |
|  | S3 | 845 | 802 | 43 | 1.15 | 1.04 | .063 | Better / Good |
| **IG Predators** |  | | | | | | | |
|  | S1 | 900 | 862 | 38 | 1.11 | 1.02 | .125 | Similar / Good |
|  | S2 | 381 | 345 | 36 | 1.14 | 1.04 | .070 | Better! / Very Good |
|  | S3 | 922 | 887 | 35 | 1.07 | 1.05 | .115 | Similar / Very Good |

**S3.4 Effect Sizes: Fencing**

The same modeling approach as in S3.3, including removal of outliers, was used to estimate the effect of fencing for sampling periods S2 and S4. Treatment had only two levels: None and REF. The repeated-measure LMM and GLMM’s revealed no Treatment x Time interaction, but the effect size (None/REF) was still extracted for each sampling date for comparison.
